# Competitive Inhibition and Pathway Truncation During Biotransformation of Traditional and Novel Brominated Flame Retardants Using a Dehalogenimonas-Rich Consortium: Chemical and Microbiological Insights

**DOI:** 10.3390/ijms27146379

**Published:** 2026-07-17

**Authors:** Yukai Zhang, Chenchen Huang, Yin-E Liu, Zhuo Wang, Tihang Wang, Yanting Zhang, Qihong Lu, Yanhong Zeng, Shanquan Wang, Bixian Mai

**Affiliations:** 1 School of Environmental Science & Spatial Informatics, China University of Mining & Technology, Xuzhou 221116, China; 2 State Key Laboratory of Advanced Environmental Technology, Guangzhou Institute of Geochemistry, Chinese Academy of Sciences, Guangzhou 510640, China; 3 University of Chinese Academy of Sciences, Beijing 100049, China; 4 School of Environmental Science and Engineering, Sun Yat-sen University, Guangzhou 510006, China; 5 Guangdong-Hong Kong-MaCao Joint Laboratory for Environmental Pollution and Control, Guangzhou Institute of Geochemistry, Chinese Academy of Sciences, Guangzhou 510640, China

**Keywords:** reductive debromination, polybrominated diphenyl ethers, 1,2,5,6-tetrabromocyclooctane, *Dehalogenimonas*, co-exposure

## Abstract

The ubiquitous co-contamination of traditional and novel brominated flame retardants (TBFRs and NBFRs) in anaerobic environments necessitates a comprehensive understanding of their combined environmental fate. This study investigated the anaerobic biotransformation of BDE 99 (a legacy aromatic TBFR) and *β*-TBCO (a cycloaliphatic NBFR) using a *Dehalogenimonas*-containing mixed culture (QY2-S1) under single- and co-exposure conditions. In a single-exposure system, QY2-S1 achieved efficient transformation of both substrates, with the observed first-order kinetic constants (*k_obs_*) of 1.31 ± 0.09 d^−1^ and 2.35 ± 0.13 d^−1^ for BDE 99 and *β*-TBCO, respectively. However, the corresponding *k_obs_* values decreased by approximately 3- to 5-fold in a co-exposure system, demonstrating pronounced reciprocal kinetic inhibition. Notably, while culture QY2-S1 maintained a unique *ortho*-regioselectivity for BDE 99, its stepwise debromination was truncated at the tetra-BDE stage during co-exposure. Absolute quantitative 16S rRNA sequencing identified *Dehalogenimonas* as the sole organohalide-respiring bacterium, with its abundance initially increasing in tandem with substrate transformation. However, the dehalogenation-coupled cell growth of *Dehalogenimonas* was significantly modulated by substrate type and combination, with its absolute biomass following the order of: single *β*-TBCO > co-exposure > single BDE 99. Collectively, these results suggest that the reciprocal kinetic inhibition and the truncation of the BDE 99 debromination pathway under co-exposure are collectively driven by the suppressed growth and substrate preference of *Dehalogenimonas.* This study provides critical insights for predicting ecological risks and developing bioremediation strategies for co-occurring BFRs in real-world scenarios.

## 1. Introduction

Brominated flame retardants (BFRs) are a widely utilized class of product additives employed to comply with fire safety standards and regulations, accounting for approximately one-quarter of global flame retardant production [1]. Since BFRs are primarily added to products through physical incorporation rather than chemical bonding, they can be readily released into the environment during the production, use, recycling, and disposal of these products. These BFRs are often persistent, bioaccumulative, and toxic, making them a prominent class of organic pollutants in the environment that pose serious risks to ecosystems and human health [2,3,4,5,6]. In recent years, following the gradual phase-out of traditional brominated flame retardants (TBFRs) such as polybrominated diphenyl ethers (PBDEs), the production and use of novel brominated flame retardants (NBFRs) such as tetrabromobisphenol A (TBBPA) and 1,2,5,6-tetrabromocyclooctane (TBCO), have significantly increased. Serving as major substitutes, their global annual production reaches as high as 100,000–180,000 tons [1,7,8]. Consequently, the ongoing transition of BFRs has led to the ubiquitous co-contamination of TBFRs and NBFRs across diverse environmental and biological matrices, particularly in anaerobic sediments and soils, which act as the critical reservoirs for these hydrophobic organic pollutants [8,9,10,11,12,13,14]. This complex co-contamination landscape raises significant concerns regarding the long-term ecological risks and the combined environmental fate of these co-occurring BFRs.

In anaerobic niches, the natural attenuation of BFRs is primarily driven by microbial reductive debromination, and in situ anaerobic microbial transformation has been demonstrated for several classes of BFRs in sediments, such as PBDEs and hexabromocyclododecanes (HBCDs) [12,15]. Furthermore, anaerobic microbial transformation is considered a highly promising, economical, and environmentally friendly approach for the artificial remediation of BFR-contaminated sites [16,17]. To better understand these transformation dynamics of BFRs, extensive laboratory microcosm studies have been conducted [18,19,20,21,22]. These studies reveal that the transformation behavior of BFRs is highly dependent on their carbon skeleton structures. For instance, the debromination of aromatic BFRs, such as PBDEs, proceeds progressively via hydrogenolysis, whereas cycloaliphatic BFRs, including HBCDs and TBCO, transform mainly through dibromo-elimination [20,22]. Additionally, the carbon skeleton structure may also influence their transformation mechanisms, with *β*-TBCO undergoing *β*-elimination (E2) and PBDEs following an SN2-nucleophilic substitution [18,20]. Crucially, during the microbial transformation of multiple coexisting pollutants, interactions such as synergistic or inhibitory effects, may occur among them [23,24,25]. Therefore, to comprehensively evaluate the natural attenuation behavior and bioremediation potential of TBFRs and NBFRs characterized by distinct carbon skeleton structures, it is essential to investigate their anaerobic microbial transformation dynamics under co-occurring conditions.

Additionally, studies have demonstrated that different organohalide-respiring bacteria (OHRB) may transform BFRs through distinct debromination pathways, which may be attributed to structural differences in their respective reductive dehalogenases [25,26,27]. For instance, during the debromination of PBDEs, *Dehalococcoides mccartyi* strain MB exhibits no obvious regiospecificity, whereas *Acetobacterium* sp. AG exclusively targets the *para*-bromine atoms. In contrast, *Dehalococcoides mccartyi* strains GY50 and TZ50 preferentially remove *meta*- and *ortho*-bromines before eliminating the *para*-bromines [25]. *Dehalococcoides*, *Dehalogenimonas* and *Dehalobacter* are the three most dominant genera of OHRB, having been widely detected in anaerobic environmental matrices including sediments, soils, and sewage sludge [28,29,30,31,32]. Specifically, Xu et al. found that in PBDE-debrominating enrichment cultures derived from wastewater treatment plant sludge, over 70% of the cultures were co-dominated by *Dehalococcoides* and *Dehalogenimonas*, while nearly 20% of the cultures were dominated solely by *Dehalogenimonas*, implying that *Dehalogenimonas* has an important and often overlooked role in the debromination of BFRs [31]. Nevertheless, current research regarding the anaerobic microbial transformation of BFRs has largely revolved around *Dehalococcoides*, while the transformation behavior of these compounds mediated by *Dehalogenimonas* remains poorly understood, thereby hindering a comprehensive understanding of their anaerobic transformation in field environments [17,25,33,34].

Therefore, to bridge these critical research gaps, the anaerobic microbial transformation behaviors of two representative BFRs, BDE 99 (a legacy aromatic TBFR) and *β*-TBCO (a cycloaliphatic NBFR) mediated by a mixed culture containing *Dehalogenimonas* strains, were systematically investigated under both single- and co-exposure conditions. In this study, the microbial debromination kinetics of these two representative BFRs were quantitatively evaluated, followed by the elucidation of their debromination pathways through product qualification and quantification. Furthermore, an integrated approach combining absolute quantification and high-throughput sequencing was employed to profile the dynamic succession of key functional taxa and syntrophic networks. Special attention was directed towards the impact of co-exposure on their transformation behaviors and the resulting microbial community responses. These results will provide crucial insights for predicting the environmental fate, combined ecological risks, and bioremediation trajectories of co-occurring TBFRs and NBFRs in real-world scenarios.

## 2. Results and Discussion

### 2.1. Biotransformation Kinetics of BDE 99 and β-TBCO in Different Exposure Systems

**Figure 1:**
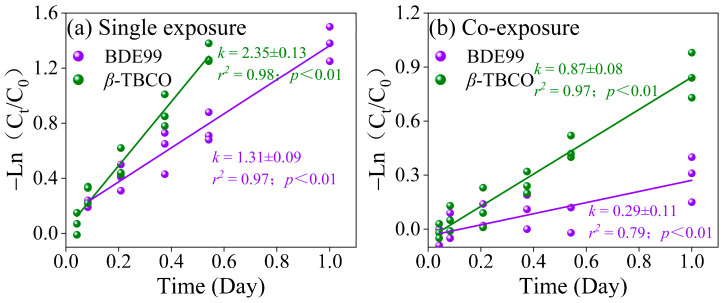
The observed first-order kinetic constants (*k_obs_*) for the anaerobic biotransformation of BDE 99 and *β*-TBCO by culture QY2-S1 under single- and co-exposure conditions.

In the sterilized control group, the concentrations of BDE 99 and *β*-TBCO remained essentially constant throughout the incubation period, with residual recoveries ranging from 95.56% to 103.75% (Figure S1). This confirms the absence of significant abiotic degradation under the tested experimental conditions. In contrast, active cultures of QY2-S1 exhibited a significant time-dependent decrease in substrate concentrations. By the end of the experiment, the residual percentages were 27.11 ± 1.85% for BDE 99 and 25.83 ± 2.41% for *β*-TBCO in single exposure system, demonstrated that culture QY2-S1 is capable of transforming both BDE 99 and *β*-TBCO. Given that the concentration profiles of both BDE 99 and *β*-TBCO in the active cultures were well-fitted to the pseudo-first-order reaction kinetic model (*r*^2^ > 0.79, *p* < 0.01), the observed first-order kinetic constants (*k_obs_*) were determined to quantify the biotransformation efficiency of the culture. Under single exposure conditions, the *k_obs_* values for BDE 99 and *β*-TBCO were 1.31 ± 0.09 d^−1^ and 2.35 ± 0.13 d^−1^, respectively (Figure 1). To date, only a few studies have characterized the anaerobic microbial transformation kinetics of BDE 99 and *β*-TBCO [18,19,21]. For instance, Lee et al. [19] reported that culture GY2 (a *Dehalococcoides* and *Desulfovibrio* consortium) achieved 88% debromination of BDE 99 (315 ± 41 nM) over 14 days. Based on their time-course data, we estimated the *k_obs_* values for BDE 99 in their system to be 0.0462 day^−1^ (0~6 days) and 0.2671 day^−1^ (6~14 days) [19]. Notably, the transformation rates achieved by culture QY2-S1 in this study significantly outperformed those of culture GY2. Similarly, the *k_obs_* values for *β*-TBCO in our study was substantially higher than values reported for the pure culture of *Dehalococcoides mccartyi* strain CG1 (*k_obs_* = 0.5232 day^−1^) and those observed in urban soil systems (0.0028~0.0184 days^−1^) [18,21]. Previous research has established that different OHRBs exhibit divergent dehalogenation efficiencies toward BFRs, such as PBDEs and TBBPA [17,23,31]. While previous studies have predominantly employed *Dehalococcoides*-based cultures, the dominant OHRB in QY2-S1 is *Dehalogenimonas*. This superior transformation efficacy is likely attributable to the distinct metabolic capabilities and specialized reductive dehalogenases (RDases) present in the *Dehalogenimonas* populations within our consortium. These findings underscore the significant potential of culture QY2-S1 for the bioremediation of sites contaminated with BDE 99 and *β*-TBCO. Furthermore, variations in dehalogenation rates across these systems can also be attributed to factors such as OHRB cell density and substrate concentration [17,33]. For instance, Xu et al. [17] reported that in sediment microcosms, the debromination rates of PBDEs were 4.2-fold and 27.4-fold higher in systems inoculated with low- and high-density strain CG1, respectively, compared to non-inoculated controls. As for substrate concentration, it not only governs the mass transfer kinetics but also modulates cellular physiological adaptation (e.g., cell membrane permeability, dehalogenase gene expression, and enzyme activity regulation), thereby shaping the overall observed biotransformation rates [35,36,37]. This is supported by findings by Zhang et al. [38], who reported significant concentration-dependent variation in the *k_obs_* values of PCB132 by strain CG1.

In field environments, various NBFRs and TBFRs with distinct carbon backbones are widely co-occurring. To elucidate the impact of co-contamination on degradation kinetics, the anaerobic microbial transformation of BDE 99 and *β*-TBCO was further investigated under co-exposure conditions. As illustrated in Figure S1, the final residual percentages in the co-exposure system were 69.89 ± 2.93% for BDE 99 and 45.46 ± 2.53% for *β*-TBCO. The corresponding *k_obs_* values were determined to be 0.29 ± 0.11 d^−1^ and 0.87 ± 0.08 d^−1^, respectively (Figure 1b). Clearly, these *k_obs_* values were significantly lower than those observed under single-exposure conditions, indicating a pronounced reciprocal inhibition between the two pollutants. This reciprocal inhibitory effect is likely attributable to the substrate competition for shared OHRBs, such as *Dehalogenimonas*, which may serve as the primary functional degraders for both substrates in this consortium. Furthermore, some research thought that the inhibition of dehalogenation may also stem from toxic effects exerted by co-existing pollutants on the physiological activity of OHRBs and their associated syntrophic consortia [39,40,41]. These reciprocal inhibitory effects are consistent with previous observations in other organohalide-respiring systems. For instance, Wang and He [34] reported a similar mutual inhibition during the co-exposure of PCBs and tetrachloroethylene (PCE) in culture AD14 (a *Dehalococcoides* and *Dehalobacter* consortium). Analogous competitive inhibition was also documented during the simultaneous transformation of 1,1,2-trichloro-1,2,2-trifluoroethane and trichloroethylene (TCE) by a *Dehalococcoides*-containing consortium [42]. These collective findings suggest that substrate competition for shared metabolic machinery is a common feature among phylogenetically diverse OHRBs when encountering complex halogenated mixtures. However, in contrast to reciprocal inhibition, several studies have documented asymmetric interactions characterized by the unidirectional promotion of one contaminant’s transformation coupled with the concurrent inhibition of the other [17,25,43]. For instance, in a strain CG1-bioaugmented sediment system, the addition of TBBPA doubled the dehalogenation kinetics of PCBs and PBDEs relative to their single-exposure controls, despite TBBPA transformation being markedly suppressed. In that scenario, TBBPA functioned as a preferential substrate that might trigger a “halogen-priming effect”. This phenomenon was driven by the stimulation of OHRB proliferation and the up-regulation of broad-spectrum reductive dehalogenase genes [33], which accelerated the biotransformation of recalcitrant PCBs and PBDEs. While reciprocal inhibition appears to be a ubiquitous feature in co-exposure systems, identifying a suitable halogen-priming agent remains a viable strategy to overcome kinetic bottlenecks and expedite the bioremediation of recalcitrant pollutants.

Notably, the *k_obs_* values of BDE 99 was significantly lower than that of *β*-TBCO in both exposure systems (Figure 1). Although the fundamental cause of this consistent kinetic discrepancy remains to be fully elucidated, inherent differences in physicochemical properties (e.g., molecular structure and activation energy) are decisive factors governing their biotransformation rates [44]. Molecular structure dictates the steric hindrance encountered during substrate binding to the dehalogenase active site, while bond dissociation energy directly influences the susceptibility of C–Br bonds to enzymatic cleavage [20,45]. Unlike aromatic BDE 99, *β*-TBCO is an aliphatic cyclic compound lacking an extensive conjugated π-system. This fundamental difference in electronic distribution inherently dictates the ease of debromination; specifically, BDE 99 undergoes debromination via a hydrogenolysis pathway, whereas *β*-TBCO proceeds through dihaloelimination (as discussed below). Similar disparities in kinetics and transformation pathways have also been documented during the biotransformation of *β*-TBCO and DPTE by strain CG1 [18]. Furthermore, the differential bioavailability and solubility between these two hydrophobic compounds likely exert additional influence on their respective transformation rates within anaerobic consortia [21,37].

### 2.2. Products and Metabolic Pathways of Single and Combined Exposures

To characterize the microbial transformation products, all peaks on the total ion chromatograms for BDE 99 and *β*-TBCO were systematically analyzed at each sampling interval using their respective mass spectra. A total of eleven debromination products were detected for BDE 99, while two metabolites were identified for *β*-TBCO.

The two *β*-TBCO metabolites exhibited identical mass spectra and very similar retention times (6.89 min and 6.99 min; Figure S2). In the absence of commercial standards, their tentative structures were elucidated based on molecular ion masses, fragment ion patterns, and characteristic bromine isotope clusters (Figure S2). These two products were identified as stereoisomers of 4,5-dibromo-9-oxabicyclo[6.1.0]nonane, which is consistent with the products detected in our previous study on *β*-TBCO degradation by strain CG1. The detailed product identification process has been published in our previous study and is also described in the Supplementary Materials. Previous studies have demonstrated that *β*-TBCO typically undergoes initial conversion to dibromocyclooctene via three distinct pathways, namely *β*-elimination (E2), Sn2-nucleophilic substitution, and homolytic substitution, followed by epoxidation to yield 4,5-dibromo-9-oxabicyclo[6.1.0]nonane (Figure S3) [18]. Incorporating dual-element isotope analysis, our prior work inferred that strain CG1 transforms *β*-TBCO through the *β*-elimination (E2) pathway. However, because different dehalogenating functional bacteria usually possess distinct dehalogenase systems, their transformation mechanisms for the same pollutant can vary significantly [46]. Consequently, based on the currently available data, it remains challenging to definitively deduce the specific mechanism governing *β*-TBCO transformation by the mixed culture QY2-S1. Additionally, although these isomers were detected in both single- and co-exposure systems, their trace concentrations and the lack of analytical standards precluded temporal quantitative analysis. This suggests a significant stoichiometric imbalance during *β*-TBCO biotransformation, indicating the formation of additional metabolites that remained undetected under current sample preparation and instrumental analytical methods.

**Figure 2:**
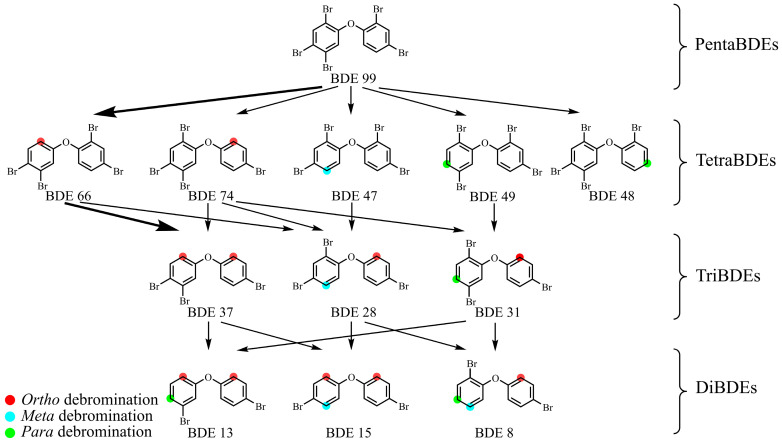
Proposed reductive debromination pathways of BDE 99 mediated by culture QY2-S1 (Note: bold lines represent the major pathways).

Regarding BDE 99, its debromination products were identified through a combination of full-scan mass spectrometry and retention time matching with commercial standards. As shown in Figure 2, the debromination of BDE 99 yielded eleven metabolites, comprising five tetra-BDEs, three Tri-BDEs and three Di-BDEs, indicating a stepwise reductive debromination process. While commercial standards were unavailable for two tetra-BDE products, their identities were elucidated through a comprehensive deduction process: since BDE 99 can theoretically generate only five tetra-BDE isomers, and BDE 47, 49, and 66 were confirmed via standards, the remaining two peaks were assigned as BDE 74 and 48. These two isomers were further distinguished based on their relative abundances and the regioselectivity of BDE 99 transformation by culture QY2-S1. Similarly, one tri-BDE product lacking a corresponding standard was tentatively identified as BDE 31 based on an analogous deductive principle.

**Figure 3:**
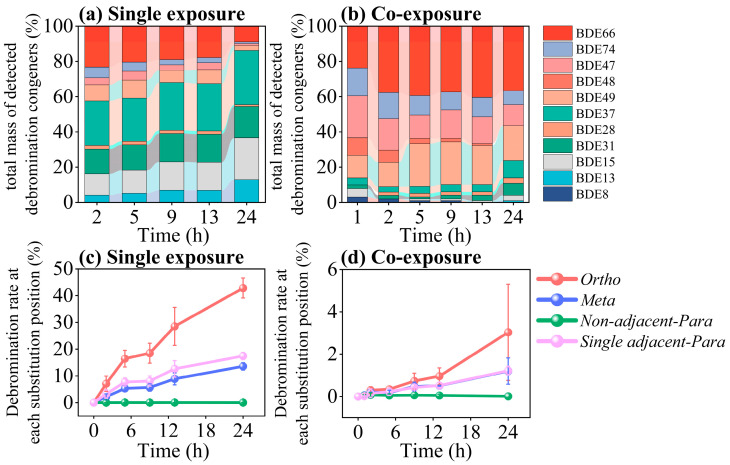
Temporal changes in product molar profiles (**a**,**b**) and regioselective bromine removal ratios (**c**,**d**) during BDE 99 debromination under single- and co-exposure conditions.

To quantitatively elucidate the debromination pathways of BDE 99 (245-24), the concentrations of individual metabolites were systematically determined. As shown in Figure 3 and Figure S4, co-exposure with *β*-TBCO significantly altered the transformation product profile of BDE 99. In the single-exposure system, the transformation was dominated by tri-BDEs; the proportions of tetra-, tri-, and di-BDEs were in the range of 14.19 ± 3.62%~42.33 ± 7.23%, 40.80 ± 0.80%~49.46 ± 1.12%, and 18.70 ± 0.38%~36.36 ± 4.73%, respectively. In contrast, tetra-BDEs became the predominant products in the co-exposure system, accounting for as much as 77.02 ± 13.77%~91.39 ± 0.01% of the total molar concentration. These findings indicate that the presence of *β*-TBCO hindered the stepwise debromination of BDE 99, causing a notable truncation of the debromination pathway. A similar phenomenon has also been observed in co-exposure systems of PBDEs and PCBs involving *Dehalococcoides mccartyi* strain MB [25]. Specifically, during the transformation of penta-BDEs by strain MB, the product profile in a single-exposure system consists of nearly equal proportions of tetra- and tri-BDEs, while co-exposure with Aroclor 1254 shifts the primary products toward tetra-BDEs. This comparison further corroborates that competitive interactions between co-existing halogenated contaminants can significantly hinder the extent of reductive dehalogenation, preventing more extensive or “deep” debromination. Interestingly, this truncation effect on PBDEs debromination was not observed in the sediment systems bioaugmented with culture CG1 under co-exposure to PBDEs, PCBs, and TBBPA, where diphenyl ether was still the predominant end-product [17]. Integrating the transformation kinetics of PBDEs across different systems reveals a clear correlation between reaction rates and debromination depths [17,25,33]. Specifically, when the transformation rate is inhibited by the presence of co-contaminants, the extent of stepwise debromination is simultaneously hindered. Conversely, when the transformation rate is enhanced, the corresponding debromination sequence remains unaffected. These findings suggest that the depth of reductive debromination is intrinsically linked to the overall metabolic flux, where kinetic suppression serves as a precursor to the truncation of the debromination pathway. Therefore, systematic co-exposure studies involving halogenated contaminants with diverse carbon skeletons and functional groups are crucial for the identification of suitable halogen-priming agents, which could facilitate the simultaneous enhancement of both dehalogenation rates and the extent of transformation.

Furthermore, although co-exposure with *β*-TBCO truncated the extent of stepwise BDE 99 debromination, it did not significantly alter the regioselective preference of the process (Figure 3). Within both single- and co-exposure systems, BDE 66 (24-34) was the predominant tetra-BDE congener, followed by BDE 49 (24-25) and BDE 47 (24-24). Among the tri-BDE metabolites, BDE 37 (34-4) emerged as the primary product, followed by BDE 31 (25-4), while BDE 15 (4-4) constituted the predominant di-BDE congener. This metabolite profile demonstrates that culture QY2-S1 exhibits a pronounced regioselectivity for *ortho*-bromine atoms, followed by the removal of singly adjacent *para-* and *meta*-bromines (BDE 99 → BDE 66 → BDE 37 → BDE 15; Figure 2). This regioselectivity was further quantitatively corroborated by the temporal variation in position-specific bromine removal proportions throughout the incubation period (Figure 3). This suggests that while *β*-TBCO suppresses the overall catalytic efficiency of the reductive dehalogenases toward BDE 99, it does not interfere with the binding orientation of the substrate within the enzyme’s active site. Most currently enriched OHRB pure cultures and consortia, similar to the degradation patterns observed for PCBs, predominantly catalyze the *para-* and *meta*-debromination of PBDEs [26,28,33,47,48]. Therefore, given that the elimination of *ortho*-substituents is widely considered a major bottleneck in the anaerobic bioremediation of PBDEs, the distinct capability of QY2-S1 holds great promise for overcoming the recalcitrance of *ortho*-substituted congeners and achieving more extensive detoxification.

Additionally, in both single- and co-exposure systems, the total molar concentrations of BDE 99 and its identified debromination products exhibited a continuous downward trend throughout the incubation period. The final total mass balance recoveries were 79.70 ± 4.03% and 75.52 ± 2.38% for single- and co-exposure systems, respectively (Figure S5). Such a significant deficit in mass balance aligns with previous studies, such as the anaerobic microbial degradation of PBDEs in wetland bottom water and the dechlorination of PCBs by strain CG1 [20,49]. This stoichiometric imbalance strongly suggests the formation of additional metabolites that remained undetected under the current sample preparation and instrumental analytical methods, such as diphenyl ether.

### 2.3. Characteristics of Microbial Communities Under Different Exposure Systems

To elucidate the underlying microbial mechanisms governing reciprocal inhibition of dehalogenation and pathway truncation under co-exposure conditions, absolute quantitative 16S rRNA amplicon sequencing was employed. Rarefaction curves confirmed that the sequencing depth was sufficient to capture the full bacterial diversity within the QY2-S1 consortium (Figure S6). Notably, neither *α*-diversity (ACE, Chao1, and Shannon indices) nor *β*-diversity (PCoA) was significantly altered by single or combined exposure to *β*-TBCO and BDE 99 (ANOVA, *p* > 0.05; Figures S7 and S8). This ecological stability contrasts with the findings of Xu et al. [33], who reported that PBDE and PCE co-contaminants significantly shifted both the microbial diversity and community structure in strain TZ50-bioaugmented sediment microcosm, whereas only the community structure was altered in microcosms bioaugmented with strain CG1. Our results underscore the remarkable resilience of the QY2-S1 community, suggesting that OHRB-mediated dehalogenation occurs within a robust and stable ecological framework even under chemical stress. More detailed analysis revealed a consistent microbial community composition among different systems, predominantly composed of the phyla *Bacillota* (formerly *Firmicutes*; 43.84 ± 3.76%~54.15 ± 7.28%), *Chloroflexota* (19.70 ± 4.90%~29.33 ± 5.27%), and *Halobacteriota* (11.72 ± 4.57%~27.33 ± 9.08%). At the genus level, these phyla were mainly represented by *Clostridium* and *Sedimentibacter* (class *Clostridia*) as the dominant fermenters, along with *Dehalogenimonas* (class *Dehalococcoidia*) as the key functional OHRB, and *Methanoculleus* (class *Methanomicrobia*) as the primary methanogens. This robust consortium forms a synergistic metabolic network, where fermentative bacteria and methanogens provide essential electron donors (H_2_), carbon source, and other growth cofactors, required for the RDase activity of the core OHRB, *Dehalogenimonas* [50,51,52]. Consequently, while the highly abundant *Clostridium* and *Sedimentibacter* act as auxiliary fermenters, *Dehalogenimonas* is the crucial and exclusive keystone species directly responsible for the dehalogenation process. Intriguingly, while methanogens are traditionally viewed as competitors for the common electron donor (H_2_), they may indirectly facilitate organohalide respiration through interspecies hydrogen transfer. By maintaining extremely low H_2_ partial pressures, methanogens prevent the feedback inhibition of fermentation, thereby driving a steady and efficient flux of H_2_ that sustains the dehalogenation activity of *Dehalogenimonas*.

**Figure 4:**
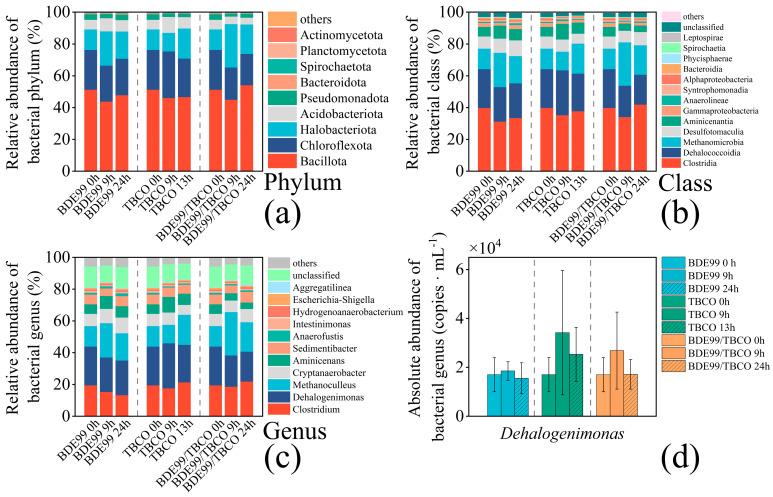
Temporal evolution of microbial community composition across various taxonomic levels and the absolute abundance of *Dehalogenimonas*. (**a**) Phylum; (**b**) Class; (**c**) Genus. (**d**) Absolute abundance of *Dehalogenimonas*. (Note: Taxa with a mean relative abundance < 0.1% for phylum and class, or <0.5% for genus, are grouped into “Others”).

Among the identified OHRB across the phyla *Bacillota*, *Chloroflexota*, and *Pseudomonadota*, only the genus *Dehalogenimonas* was detected, indicating that the debromination of both *β*-TBCO and BDE 99 was exclusively mediated by this taxon. From 0 to 9 h, a significant increase in the absolute abundance of *Dehalogenimonas* was observed in both single- and co-exposure systems (Figure 4). This rapid expansion implies that *Dehalogenimonas* likely utilizes BDE 99 and *β*-TBCO as terminal electron acceptors, coupling the dehalogenation process with energy conservation for cellular proliferation through organohalide respiration. However, from 9 h until the end of the incubation, the absolute abundance of *Dehalogenimonas* exhibited a decline across all systems (Figure 4d). This biphasic population trend, characterized by initial growth followed by a decrease, is consistent with previous reports on OHRB dynamics and may be attributed to the depletion of bioavailable substrates and the potential enhancement of toxicity from debrominated intermediates (e.g., lower-brominated BDE congeners) [17,33,47]. Notably, the absolute abundance of *Dehalogenimonas* followed the order: single-TBCO exposure > co-exposure > single-BDE 99 exposure (Figure 4). These results suggest that while *β*-TBCO is a more favorable substrate for the proliferation of *Dehalogenimonas*, its growth rate and biomass were markedly attenuated in the co-exposure system due to the inhibitory presence of BDE 99. The demographic constraint of this functional population and its substrate preference ultimately lead to the reciprocal kinetic inhibition of both contaminants and the observed truncation of the stepwise debromination pathway of BDE 99.

## 3. Methods and Materials

### 3.1. Materials

In this study, the target substrates (BDE 99 and *β*-TBCO), the surrogate standard (BDE32) and the internal standard (BDE118) were purchased from Dr. Ehrenstorfer GmbH (Augsburg, Germany) at the highest available analytical purity. A mixed standard of 39 PBDE congeners (detailed in Table S1), used for the qualitative and quantitative analysis of the substrates and debromination products, was obtained from Accustandard, Inc. (New Haven, CT, USA). The solvents, including *n*-hexane (Hex), dichloromethane, methyl *tert*-butyl ether (MTBE) and isooctane (ISO), were obtained from CNW Technologies GmbH (Düsseldorf, Germany) at HPLC grade. The debrominating enrichment culture employed herein was obtained from a closed pond severely contaminated by e-waste dismantling activities in Longtang Town (Qingyuan, China). Sediment samples were collected from depths of 6–8 cm and 12–14 cm. Each sediment sample was stored in sterilized centrifuge tubes and frozen for subsequent enrichment and cultivation of dehalogenating microorganisms. The dehalogenating microorganisms from the sediments were subjected to three to five rounds of serial enrichment in pre-prepared anaerobic flasks. PCE was added to monitor the reductive dehalogenation activity; during serial transfers, sediment impurities were gradually eliminated, yielding a clear culture suspension that retained robust reductive dehalogenation capacity. The resulting *Dehalogenimonas*-containing mixed culture QY-2 was provided by Professor Shanquan Wang’s research group at Sun Yat-sen University. After acquisition, it was re-inoculated and designated as QY2-S1. Anaerobic medium preparation and inoculation followed the protocols established by He et al. [53], with additional procedural details provided in the Supplementary Materials.

### 3.2. In Vivo Assays and Sample Pretreatment

Three anaerobic experimental groups spiked with BDE 99, *β*-TBCO, or their mixture (designated BDE 99, *β*-TBCO, and BDE 99/*β*-TBCO, respectively) were established alongside autoclaved abiotic controls. In all systems, the initial concentration of each target compound was set at 3 μM. Based on preliminary results, sampling of BDE 99-single exposure was conducted at 0, 2, 5, 9, 13, and 24 h. For the *β*-TBCO single exposure condition, samples were taken at 0, 1, 2, 5, 9, and 13 h to ensure that the parent compound conversion reached at least 80% by the final time point. In the combined exposure system, the sampling time points were set at 0, 1, 2, 5, 9, 13, 24 h. The entire experiment was conducted under strict anaerobic conditions in a constant-temperature incubator set to 30 °C in the dark. At each time point, a 5 mL aliquot was collected for pollutant analysis, and an additional 1 mL volume was reserved and stored at −80 °C for 16S rRNA gene amplicon sequencing.

The pretreatment of BDE 99 and *β*-TBCO involved inactivating the samples and adding the surrogate standard BDE32 (400 mg/L), followed by triplicate liquid–liquid extraction with a Hex/MTBE mixture (1:1, *v*/*v*). After combining the organic phases and removing residual water via anhydrous sodium sulfate, the solution was concentrated and solvent-exchanged to 10 mL of Hex. For quantitative analysis, a 0.5 mL aliquot was dried under nitrogen, reconstituted to a volume of 300 μL with ISO, and spiked with the internal standard BDE118 (5 mg/L). The remaining extract was purified using a composite neutral silica-alumina column (12:6, *v*/*v*) and concentrated to 50 μL with ISO for the qualitative identification and quantitative analysis of debromination products.

### 3.3. Instrumental Analysis

Quantitative and qualitative analyses were performed using gas chromatography–mass spectrometry (GC-MS; Agilent 8890A-5977C, Agilent Technologies, Santa Clara, CA, USA). Chromatographic separation was performed using an Agilent 19091S-433UI capillary column (30 m × 0.25 mm i.d. × 0.25 μm film thickness) with high-purity helium as the carrier gas at a constant flow rate of 1.5 mL/min. Quantitative analysis of PBDEs and *β*-TBCO was conducted in selective ion monitoring (SIM) mode using an electron ionization (EI) source. Products of PBDEs and *β*-TBCO were identified in full-scan mode and subsequently subjected to semi-quantitative analysis in SIM mode. The oven temperature program was adapted from a previous study [54], with specific settings detailed in the Supplementary Materials.

### 3.4. Microbial Community Analyses

Total genomic DNA was extracted using a commercial DNA kit according to the manufacturer’s instructions. Following extraction, DNA quality was assessed using 1.0% agarose gel electrophoresis, and the DNA concentration was quantified using a Qubit 3.0 Fluorometer (Thermo Fisher Scientific, Waltham, MA, USA). A set of 12 spike-in DNA standards was added to each sample. The hypervariable V3–V4 region of the bacterial 16S rRNA gene was amplified using the primer pair 515F (5′-GTGCCAGCMGCCGCGG-3′) and 806R (5′-GGACTACHVGGGTWTCTAAT-3′) with TransStart FastPfu DNA Polymerase (TransGen Biotech Co., Ltd., Beijing, China). The amplicons were purified, quantified, and pooled in equimolar amounts. Libraries were constructed using a TruSeq^TM^ DNA Sample Prep Kit (Illumina, San Diego, CA, USA) and sequenced on the Illumina NextSeq 2000 platform (Illumina, San Diego, CA, USA). The raw sequencing reads have been deposited in the NCBI Sequence Read Archive (SRA) database under the accession number SRP684405.

### 3.5. Quality Assurance/Quality Control (QA/QC)

Comprehensive QA/QC procedures were performed throughout the experiment. To ensure data reliability, all experiments were performed in three replicates per batch. Killed (inactivated) control groups were established alongside the active cultures for each type of exposure experiment to account for abiotic losses. For quantitative analysis, the surrogate standard BDE32 was added prior to sample extraction, yielding an average recovery of 97.60 ± 3.61% (mean ± standard deviation). BDE118 was spiked as an internal standard prior to instrumental analysis to correct for matrix effects and variations in injection volume. Solvent blanks and calibration verification standards (prepared at the same concentration as the target pollutant in the samples and spiked with the corresponding internal standards) were interspersed every 10 injections to monitor instrument response stability and potential background contamination. No target analytes were detected in the solvent blanks. Additionally, all samples were concentrated to appropriate levels prior to injection to enhance detection sensitivity. Sterile nuclease-free water was used as a negative control during DNA extraction and PCR amplification to exclude background microbial contamination.

### 3.6. Data and Statistical Analysis

The biotransformation capacity was quantitatively assessed by fitting the data to the pseudo-first-order kinetic model, yielding the rate constant (*k_obs_*), calculated as:

$$Ln(C_{t}/C_{0})=-k_{obs}\cdot t$$

where C*_t_* and C_0_ represent the substrate concentrations at time *t* and time 0, respectively. The *k_obs_* value for each substrate was obtained through linear fitting analysis, and the fit was considered statistically significant at *p* < 0.05.

Alpha diversity indices (ACE, Chao1 and Shannon) were determined via Mothur (http://www.mothur.org/wiki/Calculators, accessed on 24 November 2025) [55], while microbial community similarities were analyzed through principal coordinate analysis (PCoA) based on Bray–Curtis dissimilarity. Taxa with relative abundances below 0.1% at the phylum/class level and below 0.5% at the genus level were grouped as “others”.

## 4. Conclusions

This study demonstrates the high efficiency of the *Dehalogenimonas*-dominated mixed culture QY2-S1 in the reductive debromination of aromatic BDE 99 and cycloaliphatic *β*-TBCO. However, co-exposure of both contaminants induces potent reciprocal inhibition, reducing *k_obs_* by 3- to 5-fold relative to single-exposure scenarios. Specifically, co-existing *β*-TBCO truncates the stepwise debromination of BDE 99 at the tetra-brominated stage, whereas the unique ortho-regioselectivity of the consortium remains unaffected. This indicates that substrate competition restricts debromination depth without altering enzymatic regioselectivity. Despite the chemical stress, the QY2-S1 community exhibits high ecological resilience, as evidenced by stable *α*- and *β*-diversity. *Dehalogenimonas* is identified as the sole organohalide-respiring bacterium, and its suppressed growth and substrate preference during co-exposure serve as the key biochemical basis for the observed kinetic inhibition and pathway truncation. These results highlight that competitive interactions between structurally diverse BFRs can severely constrain the efficiency of anaerobic bioremediation, suggesting that targeted bioaugmentation or halogen-priming may be essential for enhancing the bioremediation of complex BFR-contaminated sites.

## Data Availability

The original contributions presented in this study are included in the article/Supplementary Material. Further inquiries can be directed to the corresponding author.

## Supplementary Materials

The following supporting information can be downloaded at: https://www.mdpi.com/article/10.3390/ijms27146379/s1.
