# Supplementary material for: Competitive Inhibition and Pathway Truncation During Biotransformation of Traditional and Novel Brominated Flame Retardants Using a Dehalogenimonas-Rich Consortium: Chemical and Microbiological Insights
Source: Int J Mol Sci. 2026 Jul 17;27(14):6379. doi: 10.3390/ijms27146379 (PMC13410018; doi:10.3390/ijms27146379)
Supplement: Supplementary file 1 [file ijms-27-06379-s001.zip › ijms-4367989-supplementary.pdf]

Supporting Information

Competitive Inhibition and Pathway Truncation During Biotransformation of Traditional and Novel Brominated Flame Retardants Using a *Dehalogenimonas*-Rich Consortium: Chemical and Microbiological Insights

Yukai Zhang [1], Chenchen Huang [1,2,*], Yin-E Liu [1,2], Zhuo Wang [1], Tihang Wang [1], Yanting Zhang [2,3], Qihong Lu [4], Yanhong Zeng [2,5], Shanquan Wang [4] and Bixian Mai [2,5]

[1] School of Environmental Science & Spatial Informatics, China University of Mining & Technology, Xuzhou 221116, China
[2] State Key Laboratory of Advanced Environmental Technology, Guangzhou Institute of Geochemistry, Chinese Academy of Sciences, Guangzhou 510640, China
[3] University of Chinese Academy of Sciences, Beijing 100049, China
[4] School of Environmental Science and Engineering, Sun Yat-sen University, Guangzhou 510006, China
[5] Guangdong-Hong Kong-MaCao Joint Laboratory for Environmental Pollution and Control, Guangzhou Institute of Geochemistry, Chinese Academy of Sciences, Guangzhou 510640, China
* Correspondence: huangcc@cumt.edu.cn

The supporting information contains 13 pages with 7 Figures.

Page S4: Details about the establishment of the dehalogenation microbial system.
Page S5: Details about nalytical methods and instrumental parameters for BDE99 and *β*-TBCO.
Page S6: Details about the identification process of *β*-TBCO products.

Page S8: **Figure S1.** The concentration variations of BDE 99 (a) and *β*-TBCO (b) in sterilized controls and active cultures under single and co-exposure conditions.

Page S9: **Figure S2.** Full-scan mass spectra and total ion chromatograms of *β*-TBCO dehalogenation products.

Page S10: **Figure S3.** Proposed biotransformation mechanisms of *β*-TBCO mediated by culture QY2-S1. This mechanistic scheme is inferred based on the identified intermediates (stereoisomers of 4,5-dibromo-9-oxabicyclo[6.1.0]nonane) and the previously characterized mechanism in *Dehalococcoides mccartyi* strain CG1.

Page S11: **Figure S4.** Relative molar proportions of tetra-, tri-, and di-brominated diphenyl ether congeners generated from BDE 99 under single and co-exposure conditions. panel (a) corresponds to single exposure to BDE 99; panel (b) corresponds to co-exposure with BDE 99 and *β*-TBCO.

Page S12: **Figure S5.** Mass balance of BDE 99 during anaerobic microbial transformation in two exposure systems. (ΣPBDEs: the total mass of BDE 99 and its identified debromination congeners.)

Page S13: **Figure S6.** Rarefaction curves of bacterial 16S rRNA gene sequences for QY2-S1 under different exposure conditions.

Page S14: **Figure S7.** Alpha diversity indices of the QY2-S1 consortium under single and co-exposure to *β*-TBCO and BDE 99.

Page S15: **Figure S8.** Principal coordinate analysis (PCoA) of beta diversity based on Bray-Curtis dissimilarity.

Page S16: **Table S1.** The full list of the mixed standard of 39 PBDE congeners.

Page S17: **REFERENCES**

Establishment of the dehalogenation microbial system

The preparation of the anaerobic medium and inoculation were performed following the method of He et al. [1]. The specific steps for preparing and inoculating the anaerobic medium were as follows: The inorganic salt medium was prepared according to the formula; L-cysteine and $Na_2S·9H_2O$ were added to a final concentration of 0.2 mM to maintain anoxic conditions; sodium lactate (10 mM) was supplemented as a carbon source; subsequently, the medium was autoclaved to remove oxygen, with the pH maintained at approximately 7.25. Aliquots (60 mL) of the prepared medium were dispensed into 100-mL nitrogen-purged serum bottles, which were quickly sealed with butyl rubber stoppers and secured with aluminum crimp caps to prevent gas leakage. Subsequently, the bottles were autoclaved at 121 °C for 20 minutes under 210 kPa pressure; the sterilized culture medium was colorless and clear. A vitamin storage solution (1 mL) was added to provide vitamin B_{12} for sustaining cell growth, followed by the addition of 6 μL of PCE as an electron acceptor. The serum bottles were incubated in the dark for 24 h prior to inoculation, then inoculated with a 5% (*v*/*v*) inoculum of the enriched culture stock. The cultures were then incubated in the dark at 30 °C. The PCE degradation rate was monitored in real time; when the degradation rate exceeded 80%, the culture was considered ready for use.

Instrumental parameters of GC-MS for BDE99 and *β*-TBCO

The oven temperature program was adapted from a previous study [2] and set as follows: initial temperature of 120 °C (no hold), ramped to 230 °C at 10 °C/min (held for 2 min), further increased to 260 °C at 3 °C/min (no hold), and finally ramped to 310 °C at 30 °C/min (held for 5 min). The monitored target ions were m/z 170, 248/250, 326/328, 406/408, 484/486, and 564/566. Products of PBDEs and TBCO were identified in full-scan mode and subsequently subjected to semi-quantitative analysis in SIM mode. The temperatures of the quadrupole, ion source, and injection port were maintained at 150 °C, 250 °C, and 290 °C, respectively.

The identification of β-TBCO products

Two transformation products of β-TBCO were detected in the total ion chromatogram, eluting at retention times of 6.89 min (Product 1) and 6.99 min (Product 2) (Figure S2b). Their mass spectra were essentially indistinguishable, indicating that the two products share the same molecular skeleton and were therefore assigned as stereoisomers.

The molecular ion clusters for both products were observed at m/z 282, 284, and 286, with relative intensities of 28.7%, 49.5%, and 21.7%, respectively. This distribution pattern is consistent with the natural bromine isotope abundance pattern expected for a dibrominated compound (theoretical: 30.3%:49.5%:20.3%). The fragment ion cluster at m/z 203 and 205 exhibited relative intensities of 49.1% and 50.9%, matching the characteristic pattern of a monobrominated fragment and indicating the loss of a single bromine atom from the molecular ion. Further fragmentation yielded an ion at m/z 123 that displayed no bromine-associated isotopic companion ions, suggesting the complete loss of both bromine atoms. The presence of a fragment ion at m/z 105, also observed in the mass spectrum of the parent compound β-TBCO, indicates that the cyclooctane skeleton was preserved throughout the transformation, ruling out ring-opening pathways. Notably, the molecular mass of the products (m/z 284) exceeds that of the theoretical primary dibromoelimination product, dibromocyclooctene (m/z 268), by 16 atomic mass units, which corresponds precisely to the mass of one oxygen atom. This mass difference, combined with the bromine isotope patterns and fragmentation behaviors described above, identified the two

products as stereoisomers of 4,5-dibromo-9-oxabicyclo[6.1.0]nonane. This structural assignment is supported by the identical product profile reported for *β*-TBCO transformation by *Dehalococcoides mccartyi* strain CG1 [3].

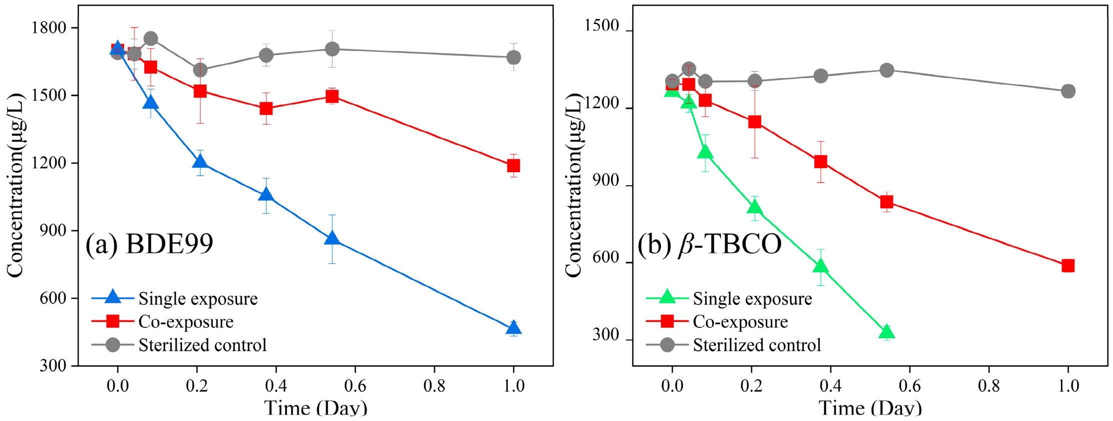

Figure S1. The concentration variations of BDE 99 (a) and *β*-TBCO (b) in sterilized controls and active cultures under single and co-exposure conditions.

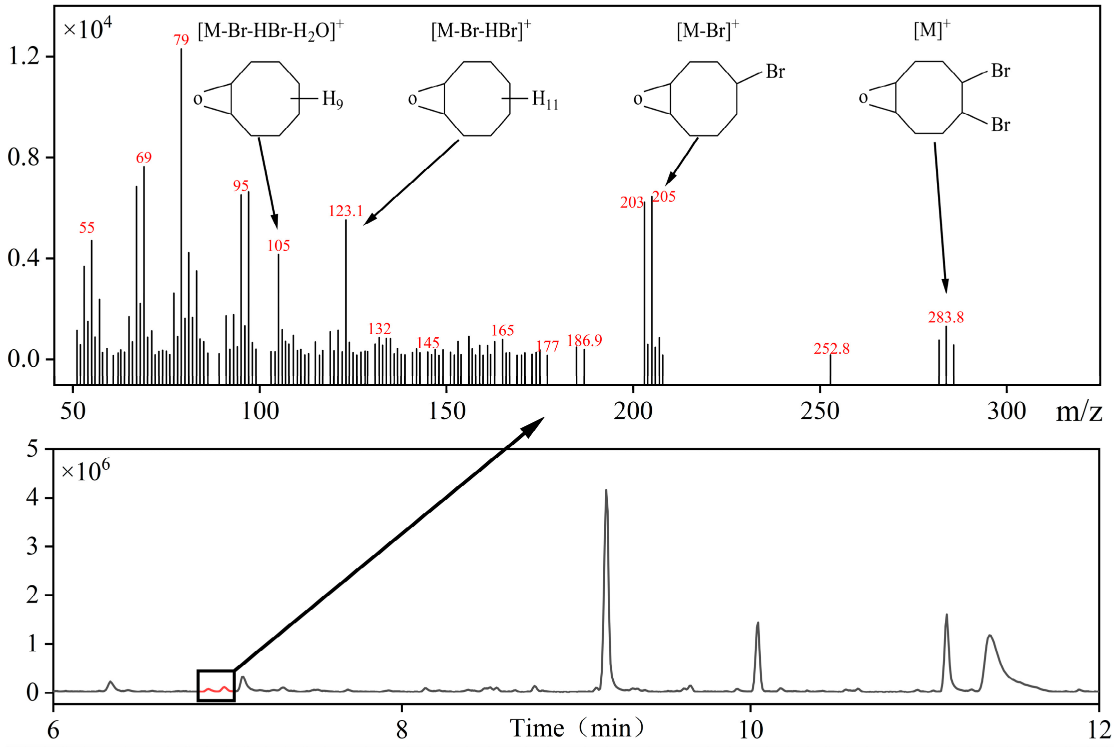

Figure S2. Full-scan mass spectra and total ion chromatograms of *β*-TBCO dehalogenation products.

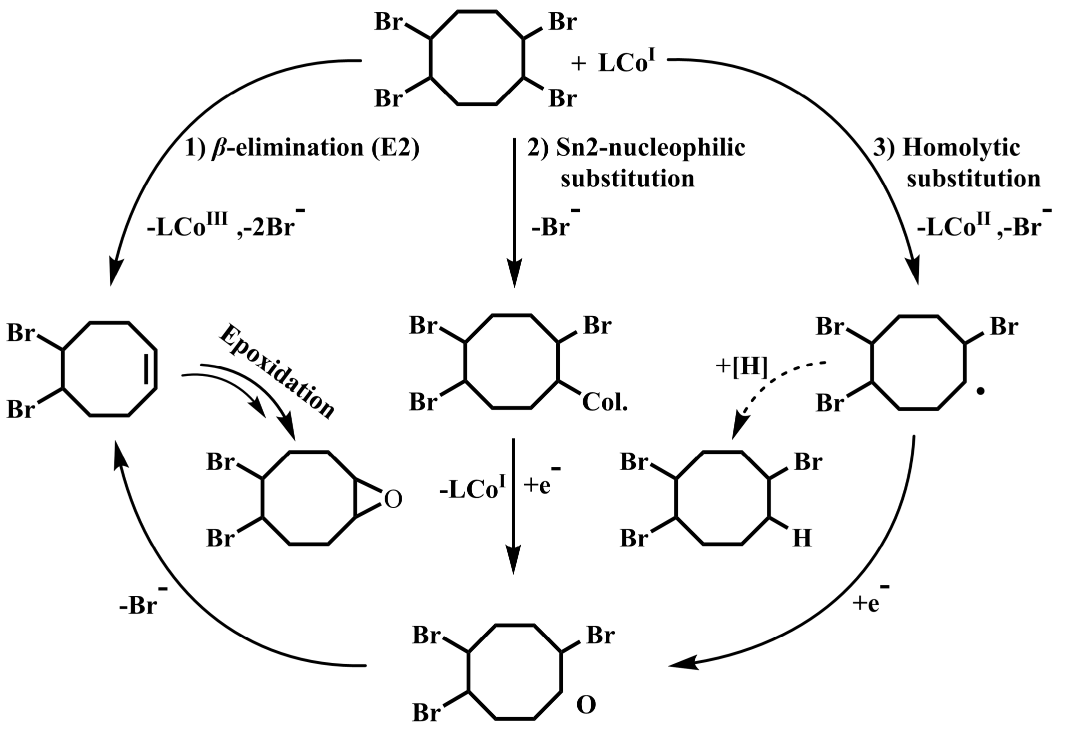

Figure S3. Proposed biotransformation mechanisms of *β*-TBCO mediated by culture QY2-S1. This mechanistic scheme is inferred based on the identified intermediates (stereoisomers of 4,5-dibromo-9-oxabicyclo[6.1.0]nonane) and the previously characterized mechanism in *Dehalococcoides mccartyi* strain CG1 [3].

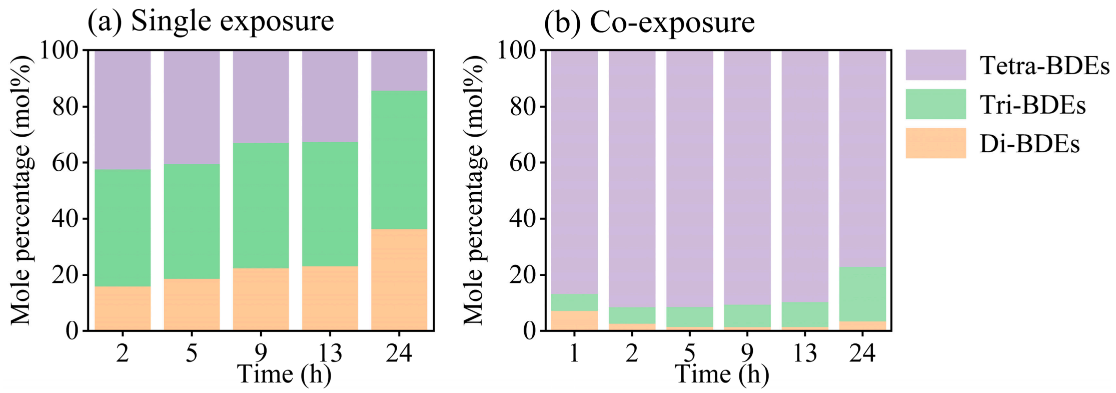

Figure S4. Relative molar proportions of tetra-, tri-, and di-brominated diphenyl ether congeners generated from BDE 99 under single and co-exposure conditions. panel (a) corresponds to single exposure to BDE 99; panel (b) corresponds to co-exposure with BDE 99 and β-TBCO.

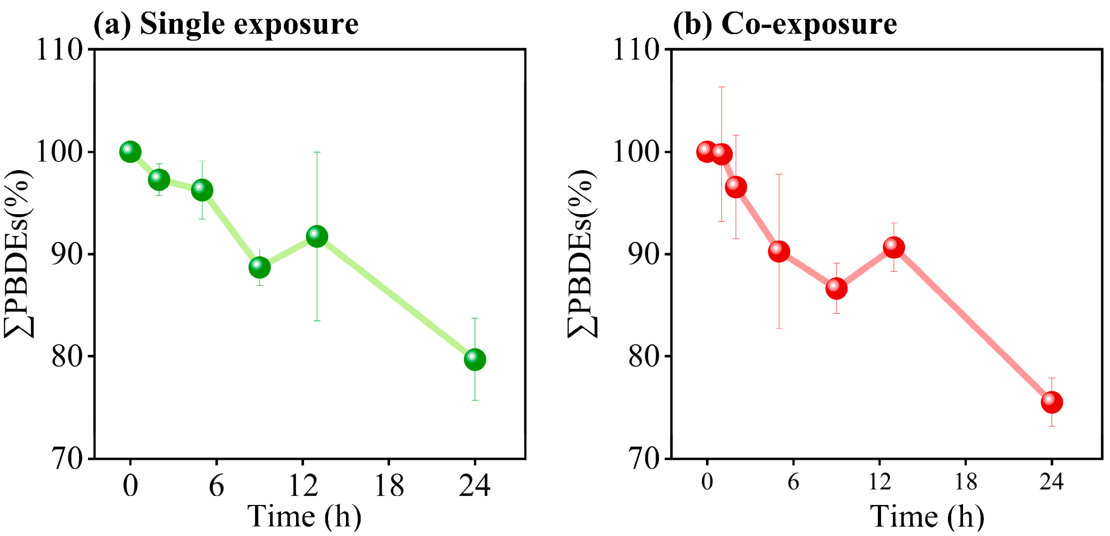

Figure S5. Mass balance of BDE 99 during anaerobic microbial transformation in two exposure systems. (∑PBDEs: the total mass of BDE 99 and its identified debromination congeners.)

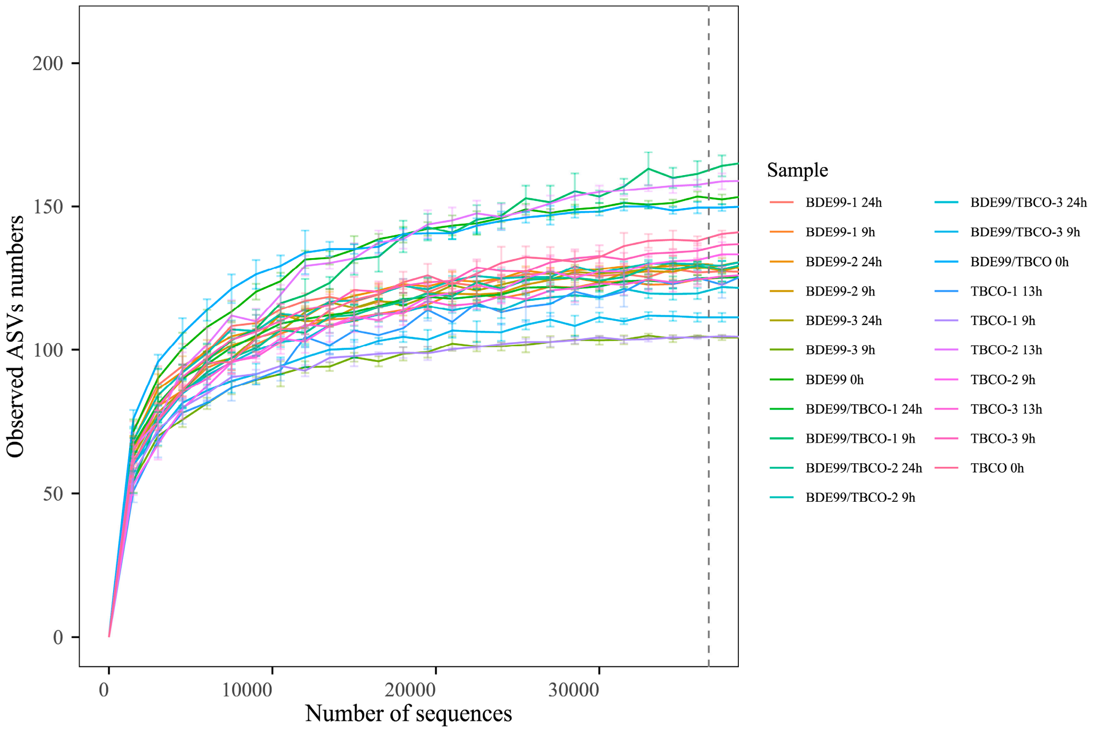

Figure S6. Rarefaction curves of bacterial 16S rRNA gene sequences for QY2-S1 under different exposure conditions.

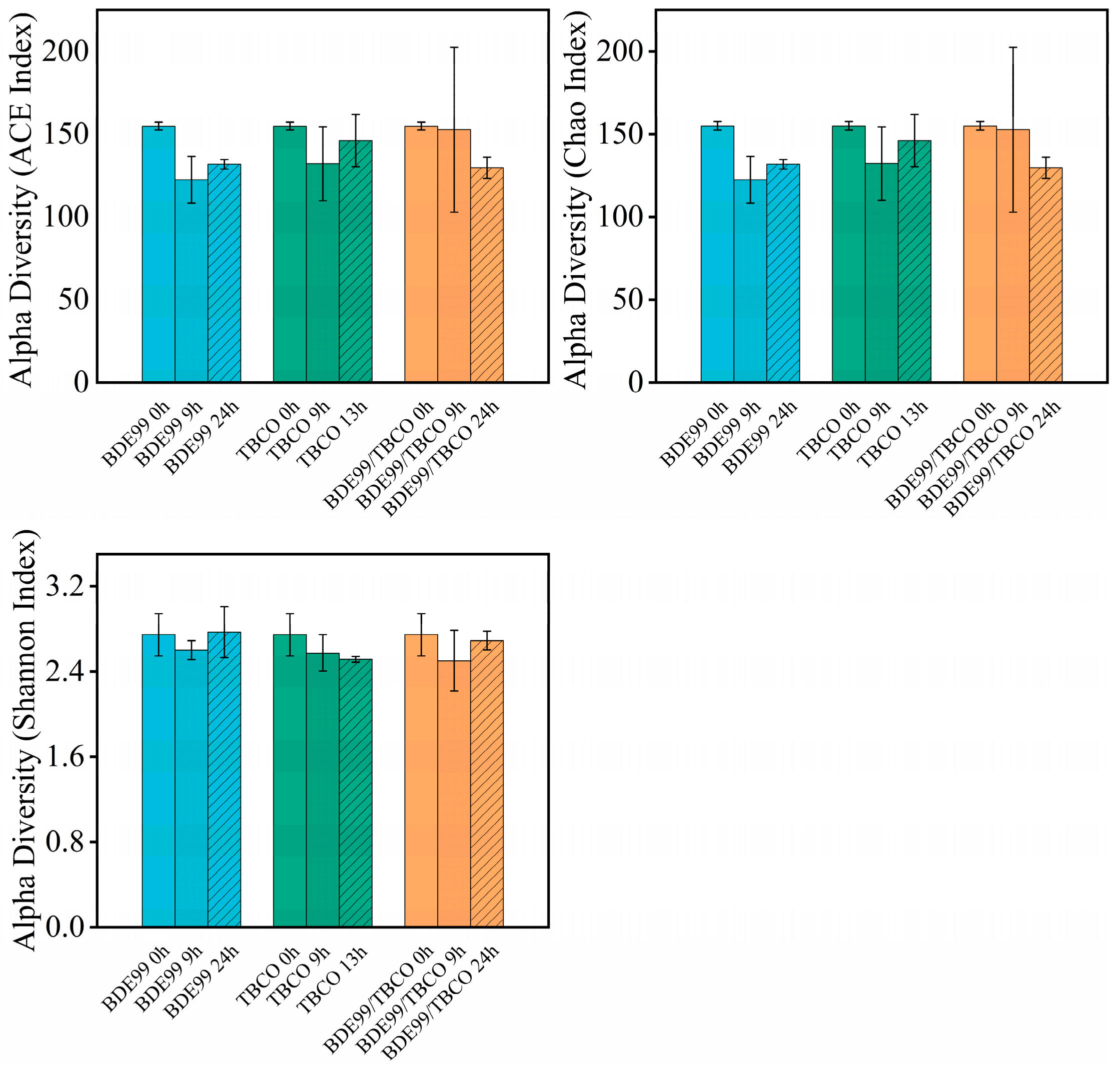

Figure S7. Alpha diversity indices of the QY2-S1 consortium under single and co-exposure to β-TBCO and BDE 99.

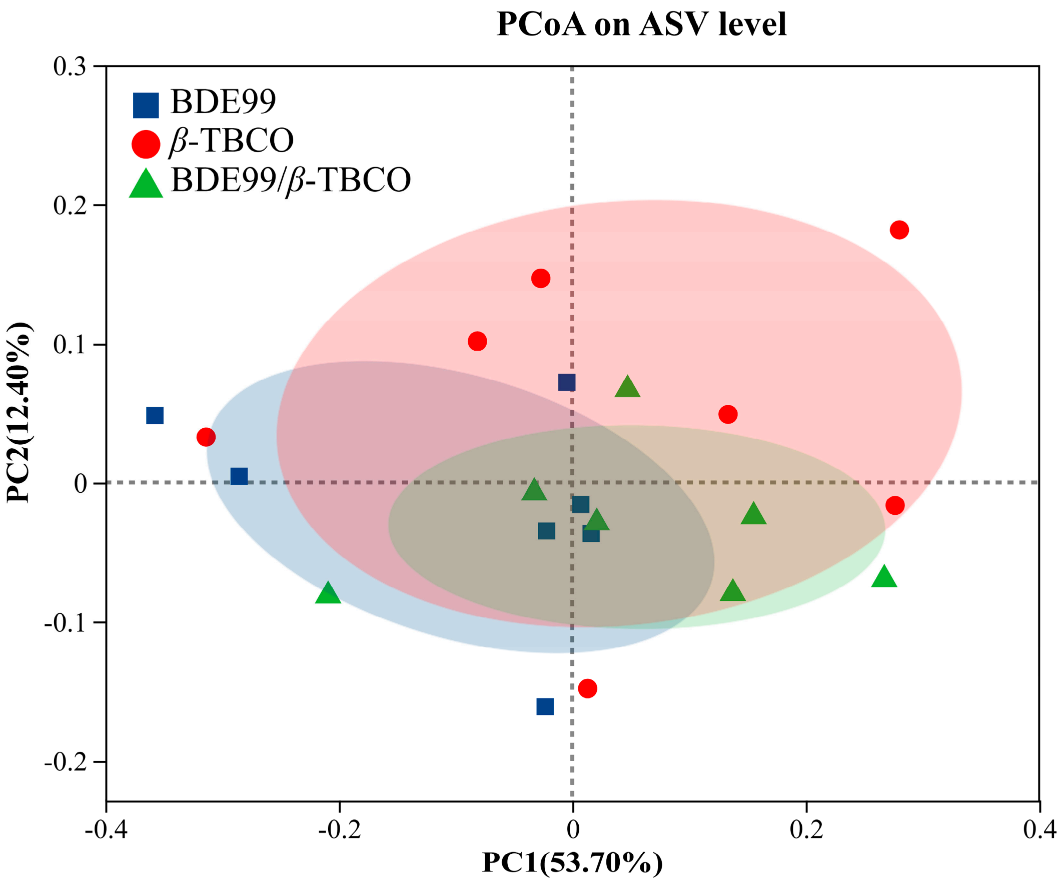

Figure S8. Principal coordinate analysis (PCoA) of beta diversity based on Bray-Curtis dissimilarity.

Table S1. The full list of the mixed standard of 39 PBDE congeners.

Mixed standard of 39 PBDE congeners					
1	BDE 1	14	BDE 25	27	BDE 99
2	BDE 2	15	BDE 33	28	BDE 116
3	BDE 3	16	BDE 28	29	BDE 118
4	BDE 10	17	BDE 35	30	BDE 155
5	BDE 7	18	BDE 37	31	BDE 85
6	BDE 11	19	BDE 75	32	BDE 126
7	BDE 8	20	BDE 49/71	33	BDE 154
8	BDE 12	21	4-F-BDE 67	34	BDE 153
9	BDE 13	22	BDE 47	35	BDE 138
10	BDE 15	23	BDE 66	36	BDE 166,
11	BDE 30	24	BDE 77	37	BDE 183
12	BDE 32	25	BDE 100	38	BDE 181
13	BDE 17	26	BDE 119	39	BDE 190

REFERENCES

1. He, J.; Ritalahti, K.M.; Yang, K.-L.; Koenigsberg, S.S.; Löffler, F.E. Detoxification of vinyl chloride to ethene coupled to growth of an anaerobic bacterium. *Nature* **2003**, *424*, 62-65, doi:10.1038/nature01717.
2. Huang, C.; Tian, Y.; Zeng, Y.; Ren, Z.; Luo, X.; Mai, B. Chlorine and Bromine Isotope Analysis of Polychlorinated Biphenyls and Polybrominated Diphenyl Ethers Using Gas Chromatography-Quadrupole Mass Spectrometry. *Journal of Chromatography. A* **2020**, *1634*, 461715, doi:10.1016/j.chroma.2020.461715.
3. Huang, C.; Zeng, Y.; Hu, K.; Jiang, Y.; Zhang, Y.; Lu, Q.; Liu, Y.-E.; Gao, S.; Wang, S.; Luo, X.; et al. Anaerobic biotransformation of two novel brominated flame retardants: kinetics, isotope fractionation and reaction mechanisms. *Water Research* **2023**, *243*, 120361-120360, doi:10.1016/j.watres.2023.120360.
